# A Bcr-Abl Inhibitor GNF-2 Attenuates Inflammatory Activation of Glia and Chronic Pain

**DOI:** 10.3389/fphar.2019.00543

**Published:** 2019-05-20

**Authors:** Gyun Jee Song, Md Habibur Rahman, Mithilesh Kumar Jha, Deepak Prasad Gupta, Sung Hee Park, Jae-Hong Kim, Sun-Hwa Lee, In-Kyu Lee, Taebo Sim, Yong Chul Bae, Won-Ha Lee, Kyoungho Suk

**Affiliations:** ^1^Department of Medical Science, College of Medicine, Catholic Kwandong University, Gangneung-si, South Korea; ^2^Translational Brain Research Center, International St. Mary’s Hospital, Catholic Kwandong University, Incheon, South Korea; ^3^Department of Pharmacology, Brain Science and Engineering Institute, BK21 Plus KNU Biomedical Convergence Program, School of Medicine, Kyungpook National University, Daegu, South Korea; ^4^Department of Neurology, The Johns Hopkins University School of Medicine, Baltimore, MD, United States; ^5^New Drug Development Center, Daegu Gyeongbuk Medical Innovation Foundation, Daegu, and VORONOI Inc., Incheon, South Korea; ^6^Department of Internal Medicine, Division of Endocrinology and Metabolism, School of Medicine, Kyungpook National University, Daegu, South Korea; ^7^KU-KIST Graduate School of Converging Science and Technology, Korea University, Seoul, South Korea; ^8^Chemical Kinomics Research Center, Korea Institute of Science and Technology, Seoul, South Korea; ^9^Department of Anatomy and Neurobiology, School of Dentistry, Kyungpook National University, Daegu, South Korea; ^10^BK21 Plus KNU Creative BioResearch Group, School of Life Sciences, Kyungpook National University, Daegu, South Korea

**Keywords:** GNF-2, c-Abl, glia, neuroinflammation, pain

## Abstract

GNF-2 is an allosteric inhibitor of Bcr-Abl. It was developed as a new class of anti-cancer drug to treat resistant chronic myelogenous leukemia. Recent studies suggest that c-Abl inhibition would provide a neuroprotective effect in animal models of Parkinson’s disease as well as in clinical trials. However, the role of c-Abl and effects of GNF-2 in glia-mediated neuroinflammation or pain hypersensitivity has not been investigated. Thus, in the present study, we tested the hypothesis that c-Abl inhibition by GNF-2 may attenuate the inflammatory activation of glia and the ensuing pain behaviors in animal models. Our results show that GNF-2 reduced lipopolysaccharide (LPS)-induced nitric oxide and pro-inflammatory cytokine production in cultured glial cells in a c-Abl-dependent manner. The small interfering ribonucleic acid (siRNA)-mediated knockdown of c-Abl attenuated LPS-induced nuclear factor kappa light chain enhancer of activated B cell (NF-κB) activation and the production of pro-inflammatory mediators in glial cell cultures. Moreover, GNF-2 administration significantly attenuated mechanical and thermal hypersensitivities in experimental models of diabetic and inflammatory pain. Together, our findings suggest the involvement of c-Abl in neuroinflammation and pain pathogenesis and that GNF-2 can be used for the management of chronic pain.

## Introduction

Neuroinflammation is highly associated with several neurodegenerative diseases including Alzheimer’s disease (AD), Parkinson’s disease (PD), and chronic pain (Katsulov and Mazneikova, 1987; Mosley et al., 2006; Tansey et al., 2007; Calsolaro and Edison, 2016; Chen et al., 2018). The chronic pain pathophysiology is complex and includes peripheral and central neuronal alterations and neuroinflammation. The neuroinflammatory process is characterized by the activation of microglia and astrocytes, macrophage infiltration, release of diverse proinflammatory mediators [e.g., nitric oxide (NO), cytokines, and chemokines]. This process leads to neuronal death or neurodegeneration (Frank-Cannon et al., 2009; Song and Suk, 2017). In this regard, important clues to the molecular mechanisms of neuropathic pain may be found by closely examining the microglial inflammatory activation and neuroinflammation (Carniglia et al., 2017; Kiguchi et al., 2017; Chen et al., 2018). Therefore, studies which target inflammatory mediators may provide novel therapeutic approaches for chronic pain management.

Non-receptor tyrosine kinase c-Abl activation is associated with AD and PD pathogenesis in human and animal models. c-Abl phosphorylation is robustly increased in brain samples from AD and PD patients as well as in animal models of AD, PD, and synucleinopathies (Ko et al., 2010; Imam et al., 2011; Vargas et al., 2018). Furthermore, imatinib or nilotinib, which are FDA-approved c-Abl inhibitors, showed neuroprotective effects when administered in animal models of PD and AD (Cancino et al., 2008; Hebron et al., 2013). More recently, activated c-Abl was observed in the spinal cord of G93A-SOD1 transgenic mice, a widely-used model of amyotrophic lateral sclerosis (ALS). This study revealed that the administration of dasatinib (a c-Abl inhibitor) improved the innervation status of neuromuscular junctions (Katsumata et al., 2012). It is quite well known that oxidative stress-induced c-Abl activation leads to nuclear factor kappa light chain enhancer of activated B cell (NF-κB) activation and neuronal death (Xiao et al., 2011). However, most studies have focused on the neuroprotective effects of c-Abl inhibitors and related molecular mechanisms in neurons. Recent findings on pain pathogenesis demonstrate that glial cells, particularly microglia and astrocytes, are an important source of inflammatory mediators fundamentally involved in the pathogenesis of inflammatory and neuropathic pain (Carniglia et al., 2017; Chen et al., 2018). Therefore, it is necessary to study the function of glial c-Abl in the pathogenesis of both inflammatory and neuropathic pain.

GNF-2 is a selective allosteric inhibitor of Bcr-Abl (the oncogenic fusion protein of Bcr and c-Abl caused by reciprocal chromosomal translocations), which was developed as an anti-cancer drug (Zhang et al., 2010; Rossari et al., 2018). GNF-2 binds to the myristate-binding site of c-Abl, leading to improved pharmacokinetic properties (Fabbro et al., 2010; Zhang et al., 2010). GNF-2 is a very selective non-ATP competitive inhibitor of Bcr-Abl and c-Abl. Unlike other inhibitors, it does not show activity against many other kinases such as fms-like tyrosine kinase 3, platelet-derived growth factor receptor, Janus kinase-1, tyrosine-protein kinase Met. Therefore, in this study, we used GNF-2 to assess the effect of c-Abl on neuroinflammation and associated pain pathogenesis using multiple pain models. It has been reported that reactive microglia release a various array of toxic molecules including pro-inflammatory cytokines, NO, and superoxide, which have been shown to play a complex role in the pathogenesis of neuropathic pain. However, the effects of c-Abl inhibition by GNF-2 on neuroinflammation and associated chronic pain pathogenesis remain elusive. Thus, in the present study, we investigated the role of c-Abl in the inflammatory activation of glia and their contribution to the pathogenesis of inflammatory and neuropathic pain by the *in vitro* and *in vivo* application of the Bcr-Abl inhibitor GNF-2.

## Materials and Methods

### Materials

GNF-2 and methylated GNF-2 compounds were prepared as described previously (Adrian et al., 2006). Lipopolysaccharide (LPS) was purchased from Sigma-Aldrich. It was obtained from *Escherichia coli* 0111:B4 prepared by phenolic extraction and gel filtration chromatography. Recombinant mouse interferon-γ (IFN-γ) protein was purchased from R&D Systems. The c-Abl siRNA (1:1 mix of siRNA #2 and #3) and control siRNA were purchased from Genolution Pharmaceuticals (Seoul, South Korea); siCont- 5^′^-CCUCGUGCCGUUCCAUCAGGUAGUU-3^′^, siAbl-#2, 5^′^-GCAACAAGCCCACUAUCUAUU-3^′^, siAbl-#3, 5^′^-UGAUGAAGGAGAUCAAACAUU-3^′^.

### Cell Culture

BV-2 immortalized murine microglial cell line was maintained in Dulbecco’s modified Eagle’s medium (DMEM) containing 5% heat-inactivated fetal bovine serum (FBS) and 50 mg/ml gentamicin at 37°C. For mouse primary mixed glial cells (MGCs) culture, the brains of 3-day old C57BL6 mice were isolated and homogenized and mechanically disrupted by a nylon mesh. The MGCs were seeded in poly-L-lysine-coated culture flasks with DMEM containing 10% FBS, 100 U/ml of penicillin, and 100 μg/ml of streptomycin (Gibco, Grand Island, NY, United States) and allowed to grow at 37°C in a humidified atmosphere with 5% CO_2_. Culture medium was changed initially after 5 days and then changed every 3 days. After 14 days of culture, MGCs (mixed microglial and astrocytes) were prepared by trypsinization, as previously described (Song et al., 2016). The collected cells were further plated using the same media condition and used for experiments.

### Nitric Oxide Production

The BV-2 cells (4 × 10^4^ cells/well in 96-well plates) were treated with 100 ng/ml of LPS and the level of NO production was assessed by measuring the amount of nitrite as previously described (Lee et al., 2009). Briefly, After 24-h of incubation, 50 μl of the cell culture media was mixed with an equal volume of a Griess reagent (0.1% naphthylethylenediamine dihydrochloride and 1% sulfanilamide in 5% phosphoric acid) in a 96-well microtiter plate. Absorbance at 540 nm was measured on a microplate reader. Sodium nitrite was used as the standard curve to calculate NO concentration.

### Assessment of Cell Viability

Both BV-2 microglia and primary MGCs (4 × 10^4^ cells/well in 96-well plates) were used to measure cell viability using 3-(4, 5 dimethylthiazol-2-yl)-2, 5-diphenyltetrazolium bromide (MTT; Sigma-Aldrich) assay, as previously described (Song et al., 2016). After 24 h of LPS treatment, the culture media was removed and MTT (0.5 mg/ml in PBS) was added to the cells, which were then incubated at 37°C for 2 h in a 5% CO_2_ incubator. The insoluble formazan crystals were completely dissolved in DMSO. The absorbance at 570 nm was measured using a microplate reader.

### Enzyme-Linked Immunosorbent Assay (ELISA) for TNF-α

The BV-2 cells or primary cells were treated with LPS either in the presence or absence of GNF-2 for 24 h. The concentration of TNF-α protein in the culture media was assessed using a rat monoclonal anti-mouse TNF-α antibody (capture antibody), and a goat biotinylated polyclonal anti-mouse TNF-α antibody (detection antibody), as described in the product manual (ELISA development reagent; R&D systems, Minneapolis, MN, United States). The recombinant TNF-α protein was used as a standard.

### Small Interfering Ribonucleic Acid (siRNA)-Mediated Knockdown of the c-Abl Gene

Cells were transfected with siRNAs using Lipofectamine^TM^ iMAX (Invitrogen, Carlsbad, CA, United States), based on the manufacturer’s instructions. The cells were used after 48 h of transfection.

### Traditional and Real-Time Reverse Transcription Polymerase Chain Reaction (RT-PCR)

Total ribonucleic acid (RNA) was extracted from the treated cells or tissues (spinal cord and brain) using TRIZOL reagent (Invitrogen, Carlsbad, CA, United States). Reverse transcription (RT) was conducted using the Superscript II reverse transcriptase (Invitrogen) and an oligo (dT) primer. Traditional PCR amplification was done using specific primer sets at 55–60°C as annealing temperature and 25–32 cycles in a C1000 Touch Thermal Cycler (Bio-Rad, Richmond, CA, United States). PCR products with ethidium bromide were electrophoresed on a 1% agarose gel, and bands were observed under ultraviolet light for analysis. Real-time PCR was performed using One Step SYBR PrimeScript RT-PCR Kit (Takara Bio, Otsu, Shiga, Japan), according to the manufacturer’s instructions, followed by detection using the ABI Prism 7000 Sequence Detection System (Applied Biosystems, California, CA, United States). Glyceraldehyde 3-phosphate dehydrogenase (GAPDH) was used as an internal control. The primer sequences were designed based on published complementary deoxyribonucleic acid (cDNA) sequences (Table 1).

**Table 1:** DNA sequences of the primers used for RT-PCR.

Target genes	Forward primer (5^′^–>3^′^)	Reverse primer (5^′^–>3^′^)
c-Abl	GAGCCTGGCCTACAACAAGT	TGTCCAGTGCATCGCTTTCT
TNF-α	CATCTTCTCAAAATTCGAGTGACAA	ACTTGGGCAGATTGACCTCAG
IL-1β	GCAACTGTTCCTGAACTC	CTCGGAGCCTGTAGTGCA
IL-6	AGTTGCCTTCTTGGGACTGA	TCCACGATTTCCCAGAGAAC
GAPDH	ACCACAGTCCATGCCATCAC	TCCACCACCCTGTTGCTGTA


### Western Blotting Analysis

Cells or brain tissues were lysed in 300 μl of lysis buffer [150 mM sodium chloride, 1% Triton X-100, 1% sodium deoxycholate, 0.1% sodium dodecyl sulphate (SDS), 50 mM Tris-HCl (pH 7.5), 2 mM EDTA] containing mixture of Halt^TM^ protease and phosphatase inhibitors (1×) (Thermo Fisher Scientific). The brain tissues were individually homogenized and then centrifuged at 13,400×*g* at 4°C for 15 min. Protein concentration was determined using the Pierce BCA protein assay kit (Thermo Fisher Scientific). Bovine serum albumin was used as the standard. Proteins (20–30 μg) for each sample were separated using 12% sodium dodecyl sulfate-PAGE and transferred to polyvinylidene fluoride filter membranes (Bio-Rad) by the semi-dry electroblotting method. The membranes were blocked with 5% skim milk and incubated sequentially with the following primary antibodies against either c-Abl (rabbit monoclonal antibody, 1:1000; Santa Cruz), p-p65, p-65, p-IκB, IκB (rabbit monoclonal antibody, 1:1000; Cell Signaling), TNF-α (rat anti-mouse monoclonal antibody, 1:500; Millipore) or α-tubulin (mouse monoclonal antibody, 1:2000; Sigma-Aldrich) and horseradish peroxidase-conjugated secondary antibodies (anti-rabbit or mouse IgG antibody; Cell Signaling), followed by chemiluminescence detection (Thermo Fisher Scientific).

### Animals and Maintenance

All experiments were conducted in accordance with approved animal protocols and guidelines established by the Animal Care Committee of Kyungpook National University. All efforts were made to reduce the number of animals and their sufferings. Age-matched male C57BL/6 mice (8–10 weeks old) were supplied by Samtako Bio (South Korea). Mice were housed in the groups of three to five per cage under standard condition using a 12-h light/dark cycle (lights on 07:00–19:00) at a constant ambient temperature of 23 ± 2°C. Each individual animal was used for a single experiment.

### Neuroinflammation Model Based on Intraperitoneal LPS Injection

Lipopolysaccharide was administered to evoke neuroinflammation in mice as described previously (Jo et al., 2017). Mice were injected a single dose of vehicle or LPS (5 mg/kg) intraperitoneally. Phosphate buffered saline (PBS) was used as vehicle and administered the same volume. Mice were sacrificed 48 h after injection and brain tissues collected for further analysis.

### The Complete Freund’s Adjuvant (CFA)-Induced Chronic Inflammatory Pain Model

Chronic inflammation in mice was induced by a single dose of CFA injection, as described previously (Jha et al., 2015). Briefly, mice were gently anesthetized with 5% of isoflurane for induction and 2% for maintenance. They received CFA (30 μl, 0.5 mg/ml; Sigma-Aldrich) unilaterally in their left hind paws (ipsilateral paws) by intraplantar injections. Mice in the control group received an equal amount of saline in their left hind paws. Pain behaviors were assessed before and up to 5 days post-CFA administration.

### Measurement of CFA-Induced Paw Edema

Complete Freund’s adjuvant-induced paw edema was assessed by the measurement of paw thickness. One experimenter, who was blinded to the treatment conditions, handled and tested all the animals. The dorsoventral thickness of the middle portions of the hind paws were measured using a caliper, as described previously (Jha et al., 2015).

### Streptozotocin (STZ)-Induced Diabetes Model

The mouse model of diabetes was generated as described previously (Rahman et al., 2016). Briefly, type-1 diabetes was induced by an intraperitoneal administration of STZ (150 mg/kg body weight; Sigma-Aldrich), prepared in 0.1 M citrate buffer (pH 4.5). An equal amount of citrate buffer was injected into control animals. Glycaemia level was tested in blood samples collected from the tail vein 3 days post-STZ injection by using an SD CodeFree^TM^ glucometer (SD Biosensor Inc., Suwon-si, South Korea). Mice with fasting blood glucose levels over 260 mg/dl were considered diabetic and used for further study.

### Behavioral Test for Pain

Before the actual test, mice were allowed to familiarize the experimenter, testing room, and equipment for at least 1 week. Paw withdrawal thresholds (PWTs) in response to mechanical stimulations were measured at different time points following CFA and STZ injection. The mechanical sensitivity was examined using calibrated Von Frey filaments (Bioseb^TM^, Chaville, France), as described previously (Rahman et al., 2016). PWT was calculated from five consecutive withdrawal responses using Dixon’s up-down method. Thermal hyperalgesia is defined as a decrease in paw withdrawal latencies (PWL) in response to a noxious thermal stimulus. The thermal sensitivity was tested using the Hargreaves’ Plantar Test Analgesy-Meter (Ugo Basile), as previously described (Jha et al., 2015). To obtain the mean PWL values, tests were repeated at least three times and averaged with 5 min intervals between tests to avoid heat-induced sensitization. One experimenter, who was unaware of the experimental conditions, handled and examined all the animals.

### Immunohistochemistry and Histopathology

Mice were deeply anesthetized and then subjected to intracardiac perfusion-fixation through the aorta with 0.1 M PBS followed by 4% paraformaldehyde dissolved in 0.1 M PBS. The tissues were further post-fixed in the same paraformaldehyde overnight. Tissues were washed with 0.1 M PBS and cryoprotected in 30% sucrose in 0.1 M PBS overnight at 4°C. Tissues were embedded in frozen section compound (FSC 22 Clear; Leica), and a cryostat was used to prepare 20 μm-thick cross-sections for the spinal cord tissues and 30 μm-thick coronal sections for the brain tissues. Tissue sections or fixed cells were then blocked with 1% bovine serum albumin or normal serum in 0.3% Triton X-100 for 60 min at room temperature. For immunofluorescence staining, tissue sections were incubated with the following primary antibodies against c-Abl (rabbit, 1:100; Santa Cruz), Iba-1 (goat, 1:200; Novus Biologicals, Littleton, CO, United States), GFAP (mouse, 1:500; BD Biosciences), inducible nitric oxide synthase (iNOS) antibody (mouse, 1:200 dilution; BD Transduction Laboratories) or NF-κB p65 (rabbit, 1:500) overnight at 4°C, and then incubated with Cy3- or FITC-conjugated secondary antibodies (1:200; Jackson ImmunoResearch, West Grove, PA, United States). Slides were washed three times with 0.1 M PBS in 0.3% Triton X-100, and mounted with Vectashield mounting medium (Vector Laboratories, Burlingame, CA, United States) using glass cover-slips, and visualized under a fluorescence microscope (Leica Microsystems, DM2500, Wetzlar, Germany).

### Quantification and Statistical Analysis

Statistical analysis was performed using either a Student’s *t* test or a one/two-way ANOVA with Dunnett’s multiple-comparisons test using GraphPad Prism (version 5.01). Differences with *p*-values <0.05 were considered to be statistically significant. For the immunohistochemical analysis, 5–6 microscopic images were chosen randomly for statistical analysis. For the measurement of either immunofluorescence or western blot band intensities, the area of the whole image or each band was selected, and the mean intensity was measured using the ImageJ software (National Institutes of Health, Bethesda, MD, United States). The background intensity of the band was also measured and deducted from the values obtained.

## Results

### GNF-2 Inhibits LPS-Induced Inflammatory Activation of Glial Cells in Culture

**FIGURE 1:**
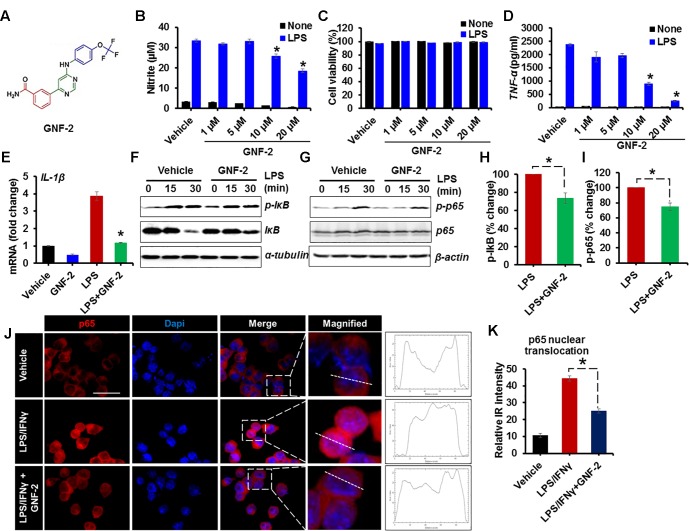
GNF-2 inhibits LPS-induced inflammatory activation of microglia. **(A)** Chemical structure of GNF-2. **(B)** The dose-dependent effect of GNF-2 on BV-2 cells in either the presence or absence of LPS (100 ng/ml) stimulation. **(C)** The cytotoxicity was measured using the MTT assay 24 h after treatment with LPS and the indicated compound concentration. **(D)** TNF-α release was measured by ELISA in BV-2 cells treated with LPS and GNF-2 for 24 h. **(E)** Real-time PCR for IL-1β mRNA expression in BV-2 cells treated with LPS or GNF-2 (10 μM) for 24 h. **(F,G)** Western blot analysis for the phosphorylation of IκB (F) or NF-kB p65 **(G)** in BV-2 cells treated with LPS for 15 and 30 min following 1 h of GNF-2 (10 μM) pre-treatment. IκB degradation was measured by anti-IκB blotting. α-tubulin and β-actin were used as loading control. **(H)** Quantification for the relative % change in p-IkB at 30 min after LPS or GNF-2 treatment. **(I)** Quantification for the relative intensity for p-p65 western blot bands at 30 min after LPS or GNF-2 treatment. **(J)** Immunocytochemistry for p65 in BV-2 cells in either presence or absence of GNF-2 with LPS treatment. Fluorescence intensity profile for p65 across a transverse section of one cell is presented adjacent to the magnified images. The dotted line shows the cross section of the single cell for the fluorescence intensity profile. **(K)** Quantification for the relative IR intensity for the p65 nuclear translocation is presented in the adjacent graph. Nuclear translocation of p65 IF intensity was measured for 10 randomly selected cells from each group with ImageJ. Data are presented as mean ± SEM. ^∗^*p* < 0.05 vs. LPS from ANOVA and unpaired two-tailed Student’s *t* test; *n* = 3 for each group. Scale bar, 50 μm.

c-Abl is activated by oxidative stress and its activation in neurons increases NF-κB activation leading to neuronal death (Xiao et al., 2011). In this study, we examined whether c-Abl is involved in the process of inflammatory microglial activation. To investigate the role of c-Abl in inflammatory microglial activation, BV-2 immortalized mouse microglial cell line was stimulated with LPS after GNF-2 pre-treatment (Figure 1A). GNF-2 significantly inhibited LPS-induced NO (Figure 1B) and TNF-α production (Figure 1D) in a dose-dependent manner and GNF-2 did not show any apparent cytotoxicity in the microglia (Figure 1C). Similarly, exposure of BV2 microglia to LPS significantly increased the expression of *IL-1β* mRNA, whereas GNF-2 treatment markedly attenuated LPS-induced upregulation of *IL-1β* (Figure 1E). GNF-2 treatment significantly reduced LPS-induced NF-κB activation in BV-2 microglial cells. Notably, LPS-induced NF-κB activation (phosphorylation of IκB and p65) was decreased in microglia following GNF-2 treatment (Figure 1F–I). NF-κB activation is associated with nuclear translocation of the p65, a component of the NF-κB complex (Tanaka and Iino, 2016). LPS-induced nuclear translocation of p65 was measured in BV-2 cells after pretreatment with GNF-2. GNF-2 significantly reduced nuclear p65 expression (Figure 1J,K). These findings suggest that GNF-2 attenuates inflammatory activation of microglia induced by LPS through inhibition of c-Abl activity.

### Knockdown of c-Abl Expression Inhibits LPS-Induced Glial Activation

**FIGURE 2:**
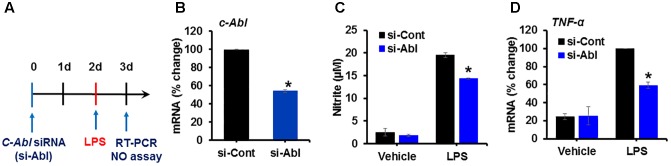
Knockdown of c-Abl expression inhibits LPS-induced inflammatory activation of microglia. **(A)** Experimental scheme. BV-2 cells were transfected with siRNA for the c-Abl gene and then treated with LPS (100 ng/ml) on day 2. Real-time PCR was performed on BV-2 cells on day 3 after transfection. **(B)** c-Abl gene expression was significantly reduced after siRNA transfection. **(C)** Nitric oxide (NO) production was measured in cells treated with LPS for 24 h after siRNA transfection. **(D)** TNF-α mRNA measured by real-time RT-PCR. si-Cont, control siRNA, si-Abl, siRNA for c-Abl gene knockdown. ^∗^*p* < 0.05 vs. si-Cont from ANOVA.

It has been documented that the expression of active c-Abl in adult mouse forebrain neurons induces severe and progressive neurodegeneration in the Cornu Ammonis 1 (CA1) region of the hippocampus and reactive gliosis (Schlatterer et al., 2011). However, the function of c-Abl in microglia upon inflammatory stimulation has not been studied yet. To examine the role of c-Abl expression in microglia following inflammatory stimulation, BV-2 cells were transfected with siRNA for c-Abl knockdown and the inflammatory activation of microglia was examined (Figure 2A,B). The knockdown of the c-Abl gene expression (more than 50%) in BV-2 cells significantly attenuated LPS-induced nitric oxide production (Figure 2C) as well as the expression of pro-inflammatory *TNF-α* mRNA (Figure 2D).

### Verification of the Anti-inflammatory Effect of GNF-2 in Primary Microglia and Astrocytes

**FIGURE 3:**
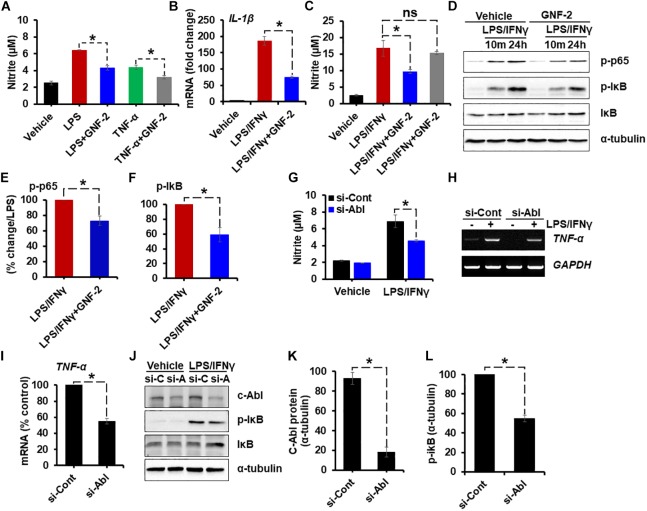
Verification of the anti-inflammatory effect of GNF-2 in primary microglia and astrocytes. Mouse mixed glial cells (MGC) were plated on 96-well plates. **(A)** Nitric oxide (NO) production induced by LPS (1 μg/ml) or TNF-α (10 μg/ml) was significantly reduced by GNF-2 treatment. **(B)** IL-1β mRNA expression induced by LPS (1 μg/ml) and IFN-γ (50 U/ml) was significantly reduced by GNF-2 treatment. **(C)** NO production induced by LPS (1 μg/ml) and IFN-γ (50 U/ml) was significantly reduced by GNF-2 treatment but not by mGNF-2, the methylated version of GNF-2. **(D)** NF-κB activation was reduced by GNF-2 pre-treatment for 1 h. Western blotting was performed to measure phosphorylation of p65 and IκB in MGC after treatment with the indicated reagents. **(E,F)** Quantification for the relative % change of p-p65 **(E)** and p-IkB **(F)** at 24 h after LPS/IFN-γ or GNF-2 treatment. **(G–L)** MGC were transfected with siRNA for the c-Abl gene and incubated for 2 days. Cells were then treated with LPS and IFN-γ for 24 h. siRNA-mediated knockdown of the c-Abl gene significantly reduced LPS/IFN-γ-induced NO production **(G)**, TNF-α mRNA expression **(H)**. **(I)** Quantification for the relative % change of TNF-α mRNA following LPS/IFN-γ treatment presented in the adjacent graph. **(J)** c-Abl expression and phosphorylation of IκB, measured by Western blot analysis. **(K)** Quantification for the relative protein expression of c-Abl after c-Abl gene knockdown following LPS/IFN-γ treatment. **(L)** Quantification for the relative protein expression for p-IkB after c-Abl gene knockdown following LPS/IFN-γ treatment. si-C, control siRNA; si-A, siRNA for c-Abl gene knockdown. Alpha-tubulin was used as the protein loading control. Data are presented as mean ± SEM. ^∗^*p* < 0.05 from ANOVA and unpaired two-tailed Student’s *t* test; *n* = 3 for each group. ns, not significant.

To determine whether the anti-inflammatory effect of GNF-2 is also observed in primary microglia and astrocytes, primary MGCs were treated with various inflammatory stimuli including LPS, TNF-α, or combination of LPS and interferon-gamma (IFN-γ). As in the BV-2 cells, GNF-2 significantly inhibited LPS-induced NO release. In addition, the anti-inflammatory effect of GNF-2 in MGC upon stimulation with recombinant TNF-α protein, an alternative potent inflammatory stimulus, was also assessed. Similarly, GNF-2 significantly inhibited TNF-α-induced NO production in primary glial cells (Figure 3A). Further, these findings led us to investigate whether GNF-2 can inhibit LPS and IFN-γ-induced production of pro-inflammatory mediators in primary glial cells or not. We found that GNF-2 pre-treatment dramatically suppressed such upregulation of *IL-1β* mRNA expression (Figure 3B). However, pre-treatment with a methylated GNF-2 analog, mGNF-2 (methylation of the aniline nitrogen at the C4 position of the pyrimidine) (Choi et al., 2009), did not suppress LPS-induced NO production. This is because methylation of GNF-2 abolished the binding specificity of GNF-2 for c-Abl (Choi et al., 2009), suggesting that the effect of GNF-2 is highly specific for c-Abl (Figure 3C). Subsequently, the effects of GNF-2 on NF-κB activation after LPS and IFN-γ stimulation was examined in MGCs pre-treated with GNF-2. The pre-exposure of MGCs to GNF-2 strongly inhibited LPS and IFN-γ–induced NF-κB activation. This was assessed by western blot analyses of phosphorylated-p65 and -IκB protein (Figure 3D–F). Furthermore, knocking down the c-Abl gene by siRNA also significantly inhibited LPS/IFN-γ-induced NO production (Figure 3G), *TNF-α* mRNA expression (Figure 3H,I), and NF-κB activation (Figure 3J–L) in the primary MGC. Taken together, these findings suggest that GNF-2 may have a potent anti-inflammatory role in glia-mediated neuroinflammation.

### Anti-inflammatory Effects of GNF-2 in a Neuroinflammation Model

**FIGURE 4:**
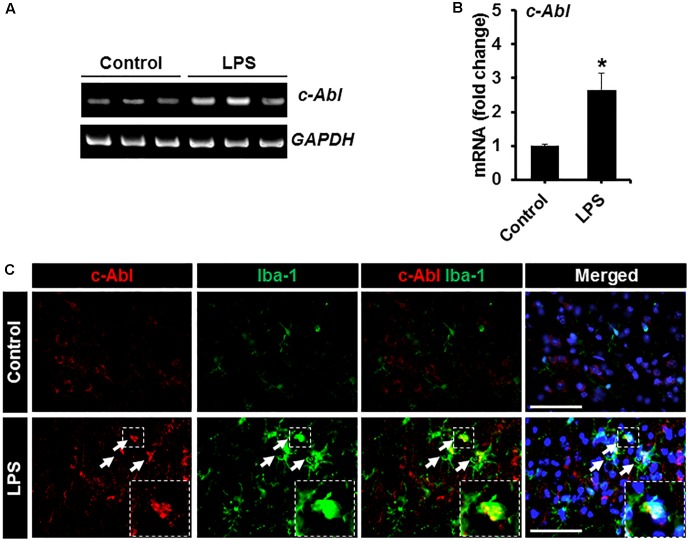
Expression of c-Abl in brain tissues after LPS injection. **(A)** The expression of c-Abl mRNA in brain tissues 48 h after the intraperitoneal injection of LPS was assessed by conventional RT-PCR. **(B)** Quantification for the c-Abl mRNA expression normalized to GAPDH. **(C)** Double immunostaining showed that c-Abl (red) expression co-localized with Iba-1 (green)-positive microglia in the cortex area of mouse brain 48 h post-LPS injection. Arrows indicate the double-labeled cells. The enlarged amoeboid shape of microglia is magnified as indicated in the dotted area. The nuclei were stained with DAPI (blue). ^∗^*p* < 0.05 vs. the vehicle-treated control animals; unpaired two-tailed Student’s *t* test; *n* = 3 for each group; data are presented as mean ± SEM. Scale bar, 100 μm.

**FIGURE 5:**
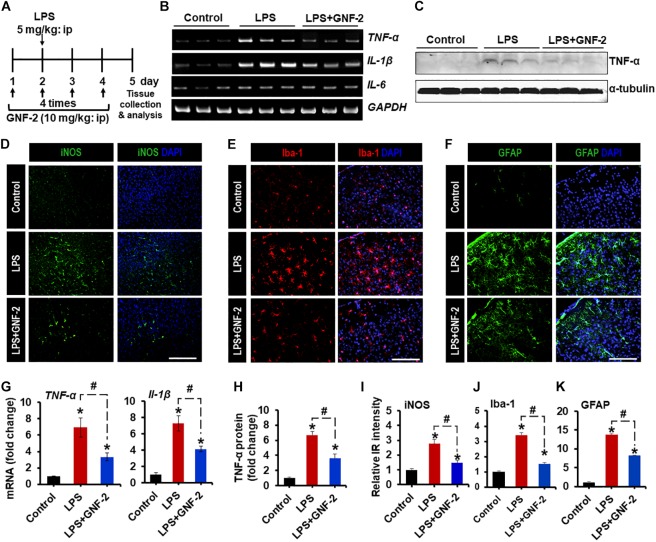
Effects of GNF-2 administration on LPS-induced neuroinflammation *in vivo*. **(A)** To determine the role of c-Abl in neuroinflammation, GNF-2, and LPS were administered intraperitoneally at the indicated time points as shown in the experimental outline. **(B)** The expression of TNF-α and IL-1β mRNAs in the brain tissues after GNF-2 and LPS injection was evaluated by conventional RT-PCR. **(C)** The western blot detection of TNF-α protein in the brain tissue after GNF-2 and LPS injection. **(D–F)** The immunoreactivity (IR) of iNOS, Iba-1, and GFAP was increased in the brain of LPS-injected mice, whereas GNF-2 administration significantly attenuated this increase in IR. The nuclei were stained with DAPI. **(G)** Quantification for the TNF-α and IL-1β mRNA expression is displayed as the fold change of gene expression normalized to GAPDH. **(H)** Quantification for the TNF-α protein from the western blot. **(I–K)** Quantification for the relative intensities of iNOS, Iba-1, and GFAP IR is presented in the graph. ^∗^*p* < 0.05 vs. vehicle-treated control animals; ^#^*p* < 0.05 between the indicated groups; unpaired two-tailed Student’s *t* test; *n* = 3 for each group; data are presented as mean ± SEM. Scale bar 400 μm **(D)**, 200 μm **(E,F)**.

To investigate the role of c-Abl in neuroinflammation, a mouse model of neuroinflammation induced by the intraperitoneal (IP) administration of LPS was used. First, the expression of c-Abl at the level of the mRNA and protein was examined by using RT-PCR and immunostaining in brain tissues isolated from LPS-injected mice. RT-PCR analysis revealed enhanced expression of *c-Abl* mRNA after 2 days post-LPS injection (Figure 4A,B). Similarly, immunofluorescence analysis showed a substantial upregulation of c-Abl protein in the brain cortex following LPS administration (Figure 4C). Co-immunostaining analysis revealed that c-Abl is found to be expressed in Iba-1-positive microglial cells, particularly in hyperactivated microglia with amoeboid shape (indicated by arrows in Figure 4C). The effect of GNF-2 on microglial activation was further tested *in vivo* using a mouse model of LPS-induced neuroinflammation. As shown in Figure 5A, GNF-2 treatment in the pre-treatment group started 24 h before LPS injection. It was administered daily for three more days. The mice were then sacrificed and examined for neuroinflammation. To evaluate the anti-inflammatory effect of GNF-2 *in vivo*, the expression of pro-inflammatory cytokines was measured in brain tissues at the levels of both mRNA and protein. The expression levels of *TNF-α* and *IL-1β* mRNA were significantly diminished following GNF-2 pre-treatment (Figure 5B,G). Similarly, GNF-2 pre-treatment significantly reduced the expression of TNF-α protein induced by LPS (Figure 5C,H). Our *in vitro* data showed a potent inhibitory effect of GNF-2 on NO production by glial cells upon inflammatory stimulation. To confirm whether GNF-2 can reduce the expression level of iNOS in the mouse brain following LPS administration, immunostaining of brain tissue sections isolated from mice treated with LPS and GNF-2 was performed. The immunostaining analyses revealed an upregulation of iNOS immunoreactivity in the brain cortex after 48 h of LPS injection when compared with vehicle-injected control animals; the immunoreactivity was significantly attenuated in GNF-2-injected mice (Figure 5D,I). Further, the levels of Iba-1 and GFAP immunoreactivity were assessed, since they are molecular markers of microglia and astrocyte activation (Rahman et al., 2016). Upon LPS injection, a significant increase in the number of Iba-1-positive microglial cells was observed in the cortex of mice brain, where microglia displayed enhanced Iba-1 immunoreactivity with short and thick processes when compared to control mice (Figure 5E,J). These morphological features of the microglia and the increased Iba-1 immunoreactivity in the cortex were attenuated in mice treated with GNF-2. Similarly, the GFAP-positive astrocytes in the cortex of LPS-injected mice showed enhanced immunoreactivity and hypertrophic morphology in comparison to that in the cortex of the vehicle-injected control animals; the immunoreactivity and hypertrophic morphology were both significantly attenuated in GNF-2-treated mice (Figure 5F,K). These findings suggest that the intraperitoneal administration of GNF-2 has a potent anti-inflammatory effect on LPS-induced neuroinflammation. The potential brain uptake mechanism of small-molecule compounds is based on physiochemical and molecular properties (Mikitsh and Chacko, 2014). In line with these parameters, GNF-2 is a small-molecule compound with comparatively low molecular weight (374.323) and has a high lipophilicity (Adrian et al., 2006), which are important criteria for BBB permeability (Mikitsh and Chacko, 2014). In addition, several studies have demonstrated that mice injected with LPS intraperitoneally show blood-brain barrier (BBB) disruption and increased permeability (Jangula and Murphy, 2013; Banks et al., 2015; Varatharaj and Galea, 2017). Thus, BBB disruption and increased permeability in the LPS model may facilitate GNF-2 transportation from circulation to the central nervous system (CNS), thereby enabling the anti-inflammatory effects of peripherally administered GNF-2 on CNS events.

### GNF-2 Ameliorates Inflammatory Pain Hypersensitivity

**FIGURE 6:**
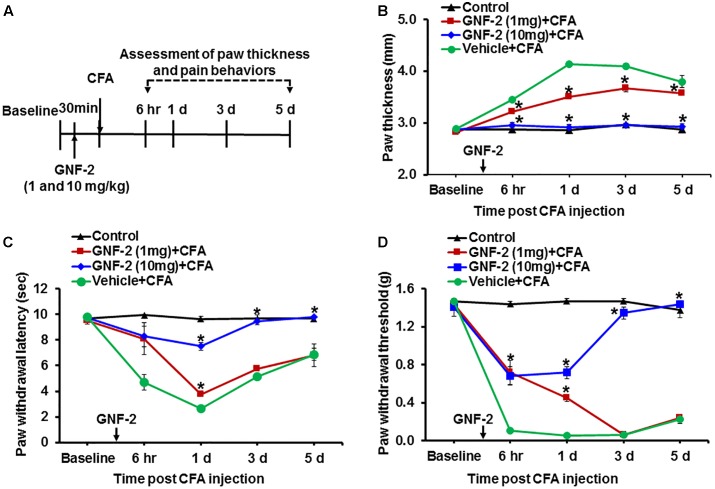
Effects of GNF-2 on CFA-induced paw edema and inflammatory pain behaviors. **(A)** To investigate the role of c-Abl in CFA-induced inflammatory pain phenotypes, GNF-2 were administered into hind paw 30 min before the intraplantar injection of CFA at the indicated time points as shown in the experimental outline. Paw edema and pain responses were measured at 6 h, 1, 3, and 5 d post-CFA injection. In the ipsilateral sides, CFA injection increased paw thickness **(B)** and reduced PWL to heat **(C)** as well as PWT to force **(D)** when compared with vehicle-injected control animals. The CFA-induced paw edema and pain hypersensitivity were attenuated in the GNF-2-injected mice (1 and 10 mg/kg) in a dose-dependent manner. No significant change in paw edema or pain-related behavior was observed in the contralateral sides and vehicle-injected animals. ^∗^*p* < 0.05 vs. vehicle + CFA injected animals Student’s *t* test; *n* = 3 for each group; data are represented as mean ± SEM. d, day (s).

Our *in vitro* and *in vivo* studies strongly indicated that treatment with GNF-2 suppresses neuroinflammation. Based on these observations, the pharmacological efficacy of GNF-2 was validated in a mouse model of CFA-induced chronic inflammatory pain. This model recapitulates several key inflammatory phenotypes including paw edema and pro-inflammatory cytokine release in the hind paw and spinal cord tissues, which has been suggested as a mechanistic consequence of peripheral and CNS pathology of CFA-induced pain hypersensitivity (Jha et al., 2014, 2016). To investigate whether intraperitoneally administering GNF-2 in this mouse model could inhibit CFA-induced pain hypersensitivity (Figure 6A), a single injection of GNF-2 (10 mg/kg body weight) was administered 30 min before CFA-administration. GNF-2-injected mice showed significantly diminished CFA (10 mg/kg)-induced paw edema formation (Figure 6B), the development of thermal hyperalgesia (Figure 6C), and mechanical allodynia (Figure 6D). However, the analgesic effect of 1 mg/kg GNF-2 was partial (Figure 6C,D). The withdrawal latency in response to thermal stimuli and the withdrawal threshold in response to mechanical stimuli were unchanged in the contralateral hind paws following CFA and GNF-2 treatment (data not shown). These results demonstrate the crucial role of c-Abl in chronic inflammatory pain.

### GNF-2 Attenuates Diabetes-Induced Neuroinflammation and Pain Hypersensitivity

**FIGURE 7:**
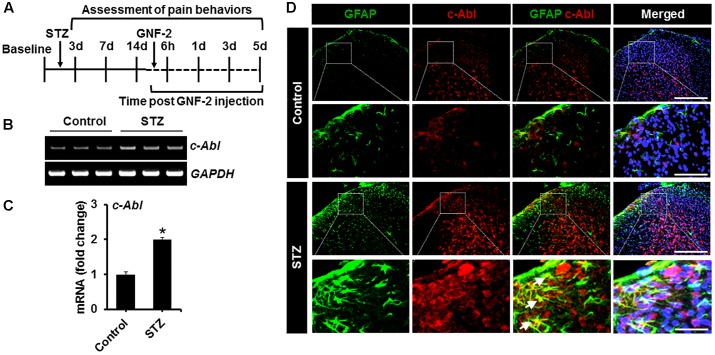
c-Abl expression in the STZ-induced neuropathic pain model. **(A)** STZ and GNF-2 were administered intraperitoneally as shown in the experimental outline. **(B)** The expression of c-Abl mRNA in the spinal cord tissues after STZ injection was evaluated by conventional RT-PCR. **(C)** Quantification for the mRNA expression is displayed as fold change of gene expression normalized to GAPDH. **(D)** Glial expression of c-Abl (arrows) was confirmed by co-staining with GFAP, a marker of astrocyte GFAP (an astrocyte marker). ^∗^*p* < 0.05 vs. vehicle-treated control animals; ^#^*p* < 0.05 between the indicated groups; unpaired two-tailed Student’s *t* test; *n* = 3 for each group; data are represented as mean ± SEM. Scale bar, 100 and 200 μm.

**FIGURE 8:**
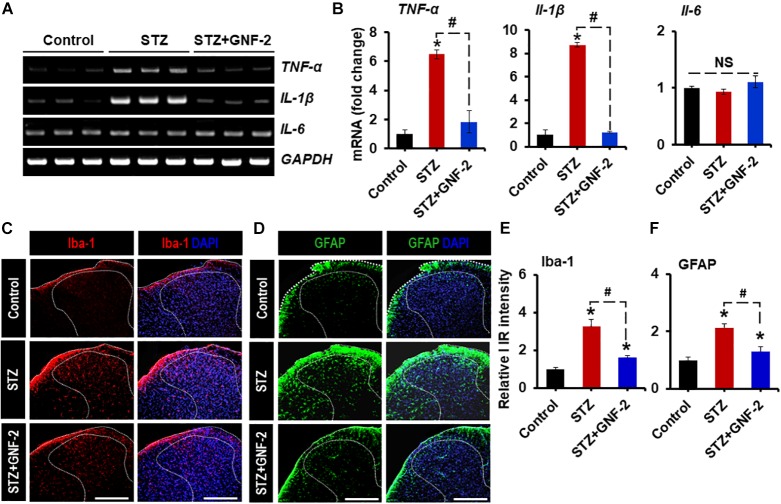
GNF-2 attenuates STZ-induced neuroinflammation. **(A)** The expression of TNF-α, IL-1β, and IL-6 mRNAs in the spinal cord tissues after GNF-2 and STZ injection was evaluated by conventional RT-PCR. **(B)** Quantification for mRNA expression is displayed as the fold increase of gene expression normalized to Gapdh. **(C,D)** Immunoreactivity (IR) of Iba-1 and GFAP was increased in the spinal cord (L4-6) of LPS-injected mice, whereas GNF-2 administration significantly attenuated this increase in IR. **(E,F)** Quantification for the relative intensity of Iba-1 and GFAP IR is presented adjacent to the microscopic images. ^∗^*p* < 0.05 vs. vehicle-treated control animals; ^#^*p* < 0.05 between the indicated groups; unpaired two-tailed Student’s *t* test; *n* = 3 for each group; data are represented as mean ± SEM. Scale bar, 100 μm.

To investigate the role of GNF-2 in neuroinflammation and pain hypersensitivity, an STZ-induced painful diabetic mouse model was also used. The mRNA and protein levels of c-Abl were first examined in spinal cord tissues isolated from mice 2 weeks after STZ injection using RT-PCR and immunostaining (Figure 7A). RT-PCR analysis revealed that the induction of diabetes significantly increases the expression of c-Abl mRNA in the spinal cord (Figure 7B,C). Similarly, immunofluorescence analysis showed a substantial upregulation of the c-Abl protein in the dorsal horn of the lumbar segment of the spinal cord (Figure 7D). In addition, c-Abl was expressed in GFAP-positive astrocytes, particularly those located in the lamina I region of the diabetic spinal cord. Subsequently, the expression levels of pro-inflammatory cytokines and glial activation were examined in the spinal cord of diabetic mice. The expression of *TNF-α* and *IL-1β* mRNAs in the lumbar segment of the spinal cord of mice with diabetes was significantly increased 2 weeks after STZ injection (Figure 8A,B). The intraperitoneal administration of GNF-2 significantly decreased the diabetes-induced increase in the expression of pro-inflammatory cytokines such as *TNF-α* and *IL-1β* mRNAs in the spinal cord tissues (Figure 8B). To evaluate the effects of GNF-2 on neuroinflammation, we also assessed diabetes-induced changes in glial activation and proliferation in the spinal cord using immunostaining. In diabetic mice, there was a significant increase in the number of Iba-1-positive microglial cells in the dorsal horn of the spinal cord tissues isolated from the lumbar segment, where the microglia displayed enhanced Iba-1 immunoreactivity with reactive morphological changes (Figure 8C). Similarly, the number of GFAP-positive astrocytes was markedly increased in the spinal cord dorsal horn of STZ-induced diabetic mice. This increase was accompanied by an increase in GFAP immunoreactivity and hypertrophic morphology with thick processes (Figure 8D). Notably, GNF-2 administration significantly downregulated Iba-1 and GFAP immunoreactivity in the spinal cord of STZ-injected mice (Figure 8E,F). These results obtained through the pharmacological inhibition of c-Abl demonstrate that c-Abl plays a crucial role in diabetes-associated neuroinflammation.

**FIGURE 9:**
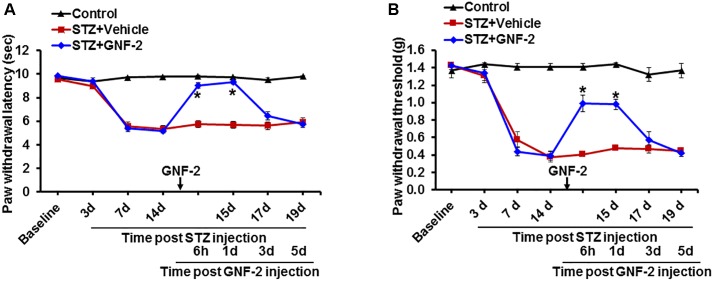
Effects of GNF-2 on STZ-induced diabetic pain. To investigate the role of c-Abl in STZ-induced pain phenotypes, GNF-2 was administered intraperitoneally at the indicated time point. STZ injection reduced PWL to heat **(A)** as well as PWT to force **(B)** when compared with vehicle-injected control animals. The STZ-induced pain hypersensitivity was attenuated in the GNF-2-injected mice (10 mg/kg). No significant change in pain-related behavior was observed in the contralateral sides and vehicle injected animals. ^∗^*p* < 0.05 vs. vehicle + STZ-injected animals Student’s *t* test; *n* = 3 for each group; data are represented as mean ± SEM. d, day (s).

Furthermore, the contribution of c-Abl activation to the pathogenesis of diabetic pain was assessed through the pharmacological inhibition of c-Abl. A single intraperitoneal injection of mice with GNF-2 (10 mg/kg) significantly attenuated diabetes-induced thermal hyperalgesia (Figure 9A) as well as mechanical allodynia (Figure 9B). However, the vehicle alone did not alter the withdrawal latency in response to thermal stimuli or withdrawal threshold in response to mechanical stimuli. These findings suggest that c-Abl plays a critical role in diabetes-induced neuroinflammation and associated pain hypersensitivity. Thus, GNF-2 might be a potent therapeutic agent for the treatment of chronic pain which results from diabetic peripheral neuropathy.

## Discussion

In this study, we show that the upregulation of c-Abl expression promotes the classical pro-inflammatory activation of microglia and that GNF-2, a potent c-Abl inhibitor, attenuates neuroinflammation and pain hypersensitivities in CFA- and STZ- induced pain models. Our findings demonstrate that c-Abl contributes to the pathogenesis of chronic pain by regulating microglial activation and neuroinflammation.

Chronic pain is caused by nerve damage which occurs during nerve compression, diabetes, inflammation, and shingles virus infection (Campbell and Meyer, 2006). Specifically, cytokines, chemokines, prostaglandins, and NO released from activated microglia and astrocytes in the dorsal horn of the spinal cord are known to play important roles in the pathogenesis of chronic pain (Skaper et al., 2012; Vega-Avelaira et al., 2013). Therefore, several studies have targeted activated microglia in order to reduce pain hypersensitivity (Hsieh et al., 2018). For example, treatment with minocycline, a microglial inhibitor, prevents pain hypersensitivity caused by systemic LPS exposure in neonates, and its protective effect may be related to its ability to attenuate LPS-induced microglial activation, pro-inflammatory cytokine IL-1β, and pain mediator PGE_2_. In addition, microglial activation is pivotal to the development and maintenance of allodynia after spinal cord injury through mechanisms involving both TNF-α and IL-1β and in chronic states, IL-6 (Detloff et al., 2008). Previous studies have shown that inflammation and oxidative stress are associated with the overexpression and activation of c-Abl (Schlatterer et al., 2011; Lawana et al., 2017). In fact, in our study, an increase in c-Abl expression was observed in the inflammatory pain models and glial activation and pain hypersensitivity were reduced by the c-Abl inhibitor.

The activation of c-Abl has been reported to play a role in neurodegenerative diseases. For example, in AD, beta-amyloid (Aβ) activates c-Abl in hippocampal neurons (Alvarez et al., 2004) and c-Abl levels are increased in pre-tangle neurons in AD (Derkinderen et al., 2005). The inhibition of c-Abl activity by Imatinib (STI-571) protects hippocampal neurons from Aβ-induced apoptosis and the reduction of c-Abl mRNA levels protects neuronal cells from Aβ-induced toxicity (Alvarez et al., 2004). Recently, it has been reported that the tyrosine phosphorylation of parkin by the oxidative stress-induced c-Abl is part of a regulatory mechanism in parkin function (Imam et al., 2011). Tyrosine phosphorylation of parkin results in impaired E3-ubiquitin ligase activity and auto-ubiquitination of parkin. c-Abl activation also plays a key role in α-synuclein neurodegeneration. c-Abl overexpression in mice leads to dopaminergic neuron degeneration and α-synuclein pathologies, while c-Abl deletion reduces α-synuclein pathologies. Activation of c-Abl leads to tyrosine 39 phosphorylation of α-synuclein, which strongly correlates with disease progression in hA53Tα-syn transgenic mice, suggesting a critical role of c-Abl in neuronal function and survival (Brahmachari et al., 2016).

The use of c-Abl inhibitors such as imatinib and nilotinib have been proposed for the treatment of AD and PD. Indeed, there have been a few reports which indicated that c-Abl inhibition might be beneficial in PD and α-synucleinopathies (Ko et al., 2010; Hebron et al., 2013). The c-Abl inhibitor, STI-571 (Imatinib), restores the E3 ligase activity of parkin and reduces the accumulation of parkin substrates, thereby protecting against 1-methyl-4-phenylpyridinium (MPP+)-induced neurotoxicity *in vitro* (Ko et al., 2010; Imam et al., 2011). However, in the aforementioned studies, it was difficult to conclude whether c-Abl inhibition could be an effective neuroprotective strategy because of the lack of selectivity of the c-Abl inhibitors they used. Imatinib and nilotinib are potent inhibitors of tyrosine kinases which include c-Abl, Src families, c-Kit, and PDGFR. However, GNF-2, a third-generation c-Abl inhibitor, had no detectable inhibitory effect on the Src family kinases Hck, Lyn, Lck, and c-Src (Choi et al., 2009). Therefore, in the current study, we used GNF-2, which is a more specific c-Abl inhibitor, and siRNA to knockdown c-Abl gene expression in order to investigate the role of c-Abl in neuroinflammation and related pathology.

## Conclusion

In conclusion, GNF-2 significantly inhibits NF-κB activation and LPS-induced pro-inflammatory molecules including TNF-α and NO in microglia and *in vivo* models of chronic inflammatory and neuropathic pain. Furthermore, we show that GNF-2 very efficiently prevents inflammatory and diabetic pain in animal models. Our results buttress the role of c-Abl in the pathogenesis of neuroinflammatory diseases. These findings indicate that c-Abl can be therapeutically targeted for both the prevention and reversal of chronic pathological pain.

## Ethics Statement

This study was carried out in accordance with the recommendations of Animal Care Committee of Kyungpook National University.

## Author Contributions

KS and GS: conceptualization. GS, MR, MJ, DG, SP, and J-HK: data acquisition and methodology. S-HL, TS, I-KL, YB, W-HL, GS, and KS: investigation. KS: project administration, resources, and supervision. GS and KS: validation. All authors wrote and edited the manuscript.

## Conflict of Interest Statement

S-HL was employed by company VORONOI Inc. The remaining authors declare that the research was conducted in the absence of any commercial or financial relationships that could be construed as a potential conflict of interest.
